# Comparative Analysis of Promoters and Enhancers in the Pituitary Glands of the Bama Xiang and Large White Pigs

**DOI:** 10.3389/fgene.2021.697994

**Published:** 2021-07-23

**Authors:** Zhimin Zhou, Yaling Zhu, Zhen Zhang, Tao Jiang, Ziqi Ling, Bin Yang, Wanbo Li

**Affiliations:** ^1^State Key Laboratory of Pig Genetic Improvement and Production Technology, Jiangxi Agricultural University, Nanchang, China; ^2^Laboratory Animal Research Center, School of Basic Medical Sciences, Anhui Medical University, Hefei, China; ^3^Key Laboratory of Healthy Mariculture for the East China Sea, Ministry of Agriculture and Rural Affairs, Jimei University, Xiamen, China

**Keywords:** ChIP-Seq, pituitary gland, pigs, H3K4me3, H3K27ac, differential peak activity, super enhancer

## Abstract

The epigenetic regulation of gene expression is implicated in complex diseases in humans and various phenotypes in other species. There has been little exploration of regulatory elements in the pig. Here, we performed chromatin immunoprecipitation coupled with high-throughput sequencing (ChIP-Seq) to profile histone H3 lysine 4 trimethylation (H3K4me3) and histone H3 lysine 27 acetylation (H3K27ac) in the pituitary gland of adult Bama Xiang and Large White pigs, which have divergent evolutionary histories and large phenotypic differences. We identified a total of 65,044 non-redundant regulatory regions, including 23,680 H3K4me3 peaks and 61,791 H3K27ac peaks (12,318 proximal and 49,473 distal), augmenting the catalog of pituitary regulatory elements in pigs. We found 793 H3K4me3 and 3,602 H3K27ac peaks that show differential activity between the two breeds, overlapping with genes involved in the Notch signaling pathway, response to growth hormone (GH), thyroid hormone signaling pathway, and immune system, and enriched for binding motifs of transcription factors (TFs), including JunB, ATF3, FRA1, and BATF. We further identified 2,025 non-redundant super enhancers from H3K27ac ChIP-seq data, among which 302 were shared in all samples of cover genes enriched for biological processes related to pituitary function. This study generated a valuable dataset of H3K4me3 and H3K27ac regions in porcine pituitary glands and revealed H3K4me3 and H3K27ac peaks with differential activity between Bama Xiang and Large White pigs.

## Introduction

Cis-regulatory elements play fundamental roles in the regulation of gene expression. Recent advances in ChIP-Seq, e.g., targeting H3 lysine 27 acetylation (H3K27ac) and H3 lysine 4 trimethylation (H3K4me3) (Barski et al., 2007), have enabled a dramatic increase in the annotations of regulatory regions, including enhancers and promoters in organisms including human, mice, and *Drosophila melanogaster* (Gerstein et al., 2010; Ernst et al., 2011; Shen et al., 2012). This has led to extensive data resources for understanding the functions of regulatory elements and molecular mechanisms underlying regulatory variants associated with diseases (Corradin and Scacheri, 2014; Zhao et al., 2018). Meanwhile, a number of studies have annotated the regulatory elements in farm animals, such as pigs (Zhao et al., 2021), cows, sheep, and chickens, coordinated by the Functional Annotation of Animal Genomes (FAANG) consortium (Andersson et al., 2015; Kern et al., 2021).

The pig is an important livestock species, serving as both a major food source and an excellent animal model for biomedical research in humans (Vodicka et al., 2005; Meurens et al., 2012). European and Chinese pigs have a divergent evolutionary history of ∼0.6 million years and were domesticated in Europe and China independently ∼9,000 years ago (Larson et al., 2005; Frantz et al., 2013). The Bama Xiang pig is a typical indigenous breed from south China, characterized by its small body size (∼60 kg at 300 days old) (Gong et al., 2019) and two-end black coat color. The Large White pig is a European breed characterized by its large and long body size (∼100 kg at 168 days old) with excellent hams, a white coat color, and a high level of reproduction; it serves as an important maternal breed for the international pork production industry (Rubin et al., 2012). The large phenotypic differences between the Bama Xiang and the Large White make them valuable animal models for comparative genetic and epigenetic studies. The pituitary gland is a master endocrine organ with an important function in regulating the growth, metabolic status, immunoregulation, reproduction, nervous system, and lactation of animals (Brinkmeier et al., 2009; Kiezun et al., 2014; Shan et al., 2014). Thus, it may play an important role in the formation of phenotypic differences between the two breeds. A previous study compared the pituitary gland transcriptome between the Bama Xiang and the Large White (Rubin et al., 2012). However, the landscapes of promoter and enhancer activity in the pituitary glands of the two breeds have not been reported.

This study profiles and characterizes the distribution of H3K4me3 and H3K27ac in the pituitary glands of two Bama Xiang and two Large White adult pigs and identifies the differential activity of H3K4me3 and H3K27ac peaks between the two breeds.

## Materials and Methods

### Samples

We carefully dissected the pituitary glands of two biological replicates (full siblings) of 150-day-old LW (one male, one female) and 132-day-old female BMX pigs, respectively, following the standardized sample collection protocols of the FAANG Project^1^ (Andersson et al., 2015). The ChIP sequencing data related to these samples were deposited at the Gene Expression Omnibus with the accession code GSE178380^2^.

### Chromatin Immunoprecipitation Sequencing

Chromatin immunoprecipitation was performed using a SimpleChIP^®^ Plus Enzymatic Chromatin IP Kit (Magnetic Beads, 9005) with 500 μg chromatin and 3 μg antibody H3K4me3 (active motif, 39,915) and 5 μg antibody H3K27ac (active motif, 39,133). The detailed protocols were obtained from https://www.encodeproject.org/about/experiment-guidelines/ and https://www.animalgenome.org/community/FAANG. The dissected tissues were treated with 37% formaldehyde to cross-link the proteins covalently with DNA. This was followed by cell disruption and sonication to shear the chromatin to a target size of 100–300 bp (Landt et al., 2012; Shen et al., 2012). Then, the modified histone with its bound DNA was enriched using the corresponding antibody. After protein removal, the DNA was purified, and real-time quantitative polymerase chain reaction (qPCR) was performed. ChIP and input (control sample) library construction and sequencing procedures were carried out according to the Illumina protocols, with minor modifications (Illumina, San Diego, CA, United States).

Trimmed clean reads were mapped to the pig reference genome *Sscrofa* 11.1^3^ using the Burrows-Wheeler Aligner (Abuin et al., 2015), allowing two mismatches and removing duplicated ChIP-seq reads using SAMtools. After that, Model-based Analysis for ChIP-Seq (MACS) version 2.1.0 peak caller was applied to infer the histone modification regions, i.e., the peaks (Zhang et al., 2008) with the following parameters: *-t Sample_ac_R1_sorted.bam -c Sample_input_R1_sorted.bam –broad -g 2.48e9 –broad-cutoff 0.1 -n Sample_ac*. The peak regions identified in individual sample were integrated by intersecting all peaks across datasets, with less than 1 kb distance between summits, using bedtools version 2.27.0 (Quinlan and Hall, 2010). We calculated the Spearman correlation coefficients of the BAM files of various ChIP-seq samples using the deepTools bamCorrelate module (Ramirez et al., 2016), and TSS enrichment analysis was performed using deepTools bamCoverge module. The library complexity was calculated using bedtools bamtobed module.

### RNA Sequencing

We downloaded RNA sequencing data (only BAM files are available) of the pituitary glands of three Bama Xiang pigs and three Large White pigs from gsa.big.ac.cn under the GSA number CRA000876^4^ (Yang et al., 2018). We extracted fastq files from the BAM files using bedtools (*bedtools bamtofastq -i sample.bam -fq sample.fq*) and remapped the fastq files to *Sus scrofa* 11.1 using STAR-2.5.3a (Dobin et al., 2013). The transcripts were assembled and merged with Stringtie with reference to Ensembl GTF (98.111), which were further merged with Ensembl GTF (98.111) to obtain a customized GTF file as the reference for the gene expression quantification. The expression levels of the genes were quantified using FeatureCounts 1.5.3 (Liao et al., 2014). After removing the mitochondrial genes, the RPKM algorithm from DESeq2 (Love et al., 2014) was used to normalize the expression values for each sample.

### Annotations of Putative Promoters and Enhancers Across Pig Genomic Features

The GAT program^5^ was used to investigate H3K4me3 and H3K27ac enrichment across genomic features with the following parameters: *gat-run.py –segment-file* = *segments.bed.gz –workspace-file* = *workspace.bed.gz –annotation-file* = *annotations.bed.gz*. The bedtools output was visualized with Integrative Genomics Viewer version 2.4, a genomic dataset viewer that allows for the visualization of genomic features. We downloaded whole genome GERP score from Ensembl Database and computed the GERP score of a peak by averaging the GERP scores of bases within that peak. GC-content of a peak was calculated using EMBOSS geecee software (Rice et al., 2000).

### Quantification of the H3K4me3 and H3K27ac Peaks and Identification of Peaks With Differential Activity Between the Two Breeds

The H3K4me3 and H3K27ac peaks were merged using the merge command in bedtools (Quinlan and Hall, 2010). The read depths in a peak region were calculated using the SAMtools bedcov utility (version 1.2) and then normalized to the RPKM value by DESeq2 R package (Love et al., 2014), where the normalized value is defined as peak activity. We further used the DESeq2 program to identify peaks to show the differential activity between the two breeds. Regions with *P* of less than 0.05 and fold changes greater than 2 were assigned as peaks with differential activity.

### Annotation and Gene Ontology Analysis of the Gene Set

Gene functions were annotated using DAVID^6^ and ClueGO (Bindea et al., 2009), and the background gene sets for enrichment analyses were all genes from the human genome. The *P* values for the enrichment of GO terms were corrected using the Benjamini-Hochberg approach. HOMER^7^ was adopted to investigate the enrichment of binding motif of transcription factors (TFs) in the corresponding set of peaks.

### Identification of Super Enhancer

The ROSE algorithm (Whyte et al., 2013) was used to identify super enhancers in all H3K27ac samples. The definition of super enhancer activity was the same as that for all peaks of H3K27ac and H3K4me3.

## Results and Discussion

### Mapping Promoters and Enhancers in the Pituitary Glands of BMX and LW Pigs

We profiled H3K4me3 and H3K27ac in the pituitary glands of two Bama Xiang (132 days old) and two Large White pigs (150 days old) using chromatin immune-precipitation, followed by high-throughput sequencing (ChIP-Seq) of single end 50 bp reads (Supplementary Figure 1A and Table 1). The average number of reads uniquely mapped to the reference genome was 25.5 M, with a range from 22.3 M to 26.8 M (Table 1). Clustering analyses performed on the BAM files based on the read depths of a set of 65,044 merged peaks first grouped the samples into two clusters represented by the two histone markers; within both clusters, the samples from the same breed were grouped together (Supplementary Figure 1B), suggesting that between-breed differences in the profiles of both H3K4me3 and H3K27ac were greater than those within breeds. PCA analyses yielded similar results (Supplementary Figure 2). We calculated the library complexity of the ChIP-seq data and obtained average Non-Redundant Fraction (NRF), PCR Bottlenecking Coefficient 1 (PBC1), and PCR Bottlenecking Coefficient 2 (PBC2) values of 0.97, 0.98, and 44.61, respectively, for the investigated samples, suggesting that the library constructions were reasonably good (Supplementary Table 1).

**TABLE 1:** Summaries of ChIP-Seq experiments.

**Samples**	**Sequencing depth**	**Total number of reads**	**Percentage of uniquely mapped reads**	**Number of peaks**	**Average peak length**	**Median peak length**
BMX_rep1_me^#^	21.49	29,083,888	89.44%	19,960	2,914	2,489
BMX_rep2_me^#^	24.58	25,373,723	88.04%	20,767	2,449	1,904
LW_rep1_me^#^	22.42	29,410,009	91.20%	17,075	3,265	3,073
LW_rep2_me^#^	24.46	27,930,694	92.90%	19,381	2,637	2,029
BMX_rep1_ac*	9.24	29,420,623	90.43%	45,464	2,973	1,555
BMX_rep2_ac*	8.22	25,238,866	90.29%	50,053	3,038	1,584
LW_rep1_ac*	9.36	29,421,070	93.29%	44,435	3,005	1,685
LW_rep2_ac*	14.00	27,881,626	93.12%	34,982	2,552	1,483

*me^#^, the abbreviation of H3K4me3, an indicative of an active promoter.*

ac*, the abbreviation of H3K27ac, an indicative of an active promoter and enhancer.

**FIGURE 1:**
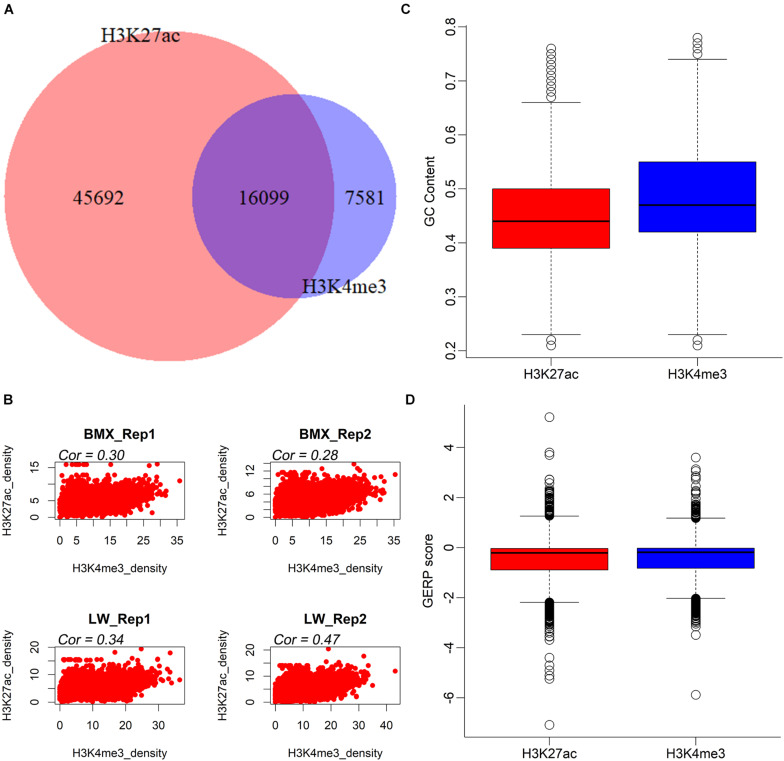
The intersection and conservation of H3K27ac and H3K4me3 peaks. **(A)** The intersection of H3K27ac and H3K4me3 peaks. **(B)** The correlation of 16,099 overlapped peaks between two histone modifications in four individuals. **(C,D)** Boxplots comparing the GC contents and GERP scores of H3K27ac and H3K4me3 peaks.

On average, we found 19,296 H3K4me3 and 43,733 H3K27ac peaks per sample (Table 1). The average/median peak lengths were 3,421/2,429 bp and 3,835/1,792 bp for the H3K4me3 and H3K27ac markers, respectively (Table 1), comparable to a previous report (Villar et al., 2015) (Supplementary Figure 3). Next, we merged all H3K4me3 and H3K27ac peaks in each individual, generating 23,680 H3K4me3 (21,396 in the BMX and the 19,553 in LW) and 61,791 H3K27ac (55,585 in the BMX and 48,333 in the LW) non-redundant peaks (Supplementary Figure 1A). The 61,791 H3K27ac peaks were further grouped into 12,138 proximal peaks that intersected with a ± 1 kb region from the transcription start site (TSS) of any transcript provided by the Ensembl database (release 98) and the remaining 49,473 distal peaks. These regions constituted a valuable resource of regulatory elements in the pituitary of the pigs. Next, we merged all H3K4me3 and H3K27ac peaks and identified 16,099 regions that overlapped with both histone modification markers, then observed significant positive correlations between the quantitative activities of the two histone modification markers across the 16,099 regions in all four individuals (r = 0.28∼0.47, *P* < 2.2 × 10^–16^) (Figures 1A,B), suggesting that H3K4me3 and H3K27ac coordinately regulated gene expression. In addition, the peak region of H3K4me3 modifications had a higher GC content and extent of sequence conservation across species (GERP score) compared to the H3K27ac peak regions (Figures 1C,D).

We assumed that the genes close to the peak with high activity may play an important function in the pituitary gland. At the 16,099 regions, we identified 100 peaks ranked within the top 5% of both histone markers in all four individuals based on their activity (see section ‘‘Materials and Methods’’). The 100 peaks overlapped with 114 genes involved in the intracellular estrogen receptor signaling pathway, positive regulation of blood pressure, and neuroepithelial cell differentiation. Among these genes, *BCL6*, *CDKN1B*, *ESRP1*, *PITX2*, and *SIX6* genes had functions related to the pituitary based on the annotation information from the DAVID bioinformatics resources^8^. For example, *BCL6* is involved in the reverse coordination of endogenous impulses released by pituitary growth hormone (GH) by interacting with *STAT5* (Meyer et al., 2009), and *CDKN1B* is associated with adreno-cortico-tropic hormone hypersecretion and the formation of pituitary neoplasms (Tichomirowa et al., 2012).

### Genomic Annotations of Putative Promoters and Enhancers

**FIGURE 2:**
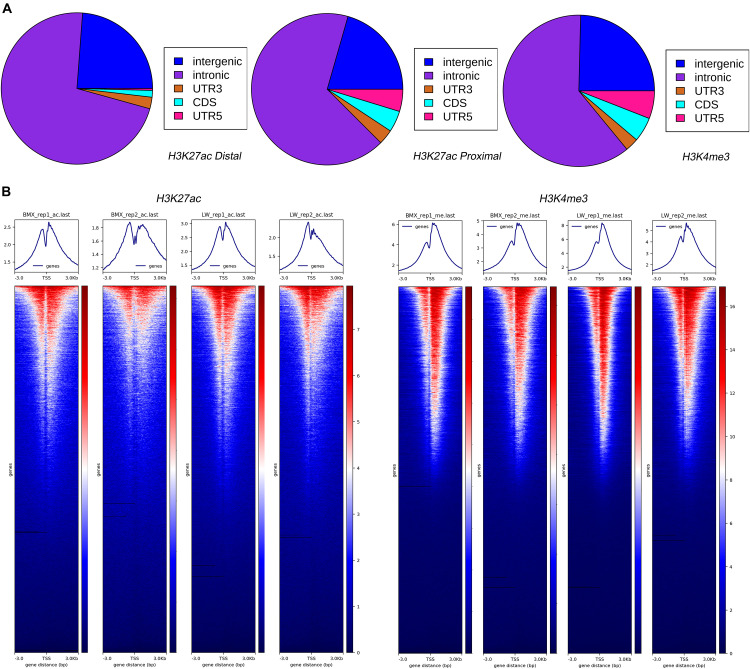
Enrichment of H3K4me3 and H3K27ac peaks across genomic features and transcription start sites. **(A)** Distribution of H3K4me3 and H3K27ac peaks across genome. **(B)** Heatmaps depicting normalized ChIP-seq signal (H3K27ac and H3K4me3) at 3 kb near the TSS, sorted by signal intensity.

Next, we examined the overlap of genomic features with the H3K4me3 and H3K27ac peaks. The H3K4me3 and H3K27ac proximal peaks were mostly located in the intron (61.36 and 66.96%) and intergenic (24.61 and 20.57%) regions, while the H3K27ac distal peaks tended to be located in intron (71.88%) and intergenic regions (23.81%) (Figure 2A). The H3K4me3 (14.03%) and H3K27ac proximal (12.47%) peaks had stronger enrichment in the transcription initiation region than the H3K27ac distal peaks (4.31%), agreeing with the fact that the promoters tend to be located near the TSS of genes, while enhancers are usually distal to the target genes (Heintzman and Ren, 2009; Pennacchio et al., 2013) (Figure 2A). This suggests that intronic sequences close to the TSS regions could play important regulatory roles. In addition, both histone modifications were enriched at 3 kb near the gene TSS, and the H3K4me3 ChIP-Seq reads showed a stronger enrichment at the TSS region than H3K27ac ChIP-Seq reads (Figure 2B). The expression levels of the genes showed significant positive correlations with the H3K4me3 and H3K27ac activity around their TSS regions (e.g., ± 5 kb) across the genome, although ChIP-Seq and RNA-Seq data (Yang et al., 2018) were generated from different samples. The correlations with gene expression were stronger for H3K4me3 than for H3K27ac. Moreover, the strengths of the correlations of the gene expressions with the ChIP-Seq peak activities decayed when greater regions centered on the TSS were examined (Supplementary Figure 4).

### Diversity of Promoter and Enhancer Peaks Among Individuals and Breeds

Next, we quantified the peak activity in each sample based on read depths in the corresponding peak region normalized by peak length, using the R package DESeq2 (see section “Materials and Methods”). We evaluated the correlation among samples in terms of genome-wide H3K4me3 and H3K27ac peak activity. The mean correlations based on H3K4me3 peak activity (0.81) were higher than those based on the H3K27ac activity (0.42), suggesting that the activity of the promoters was more conservative than those of enhancers.

**FIGURE 3:**
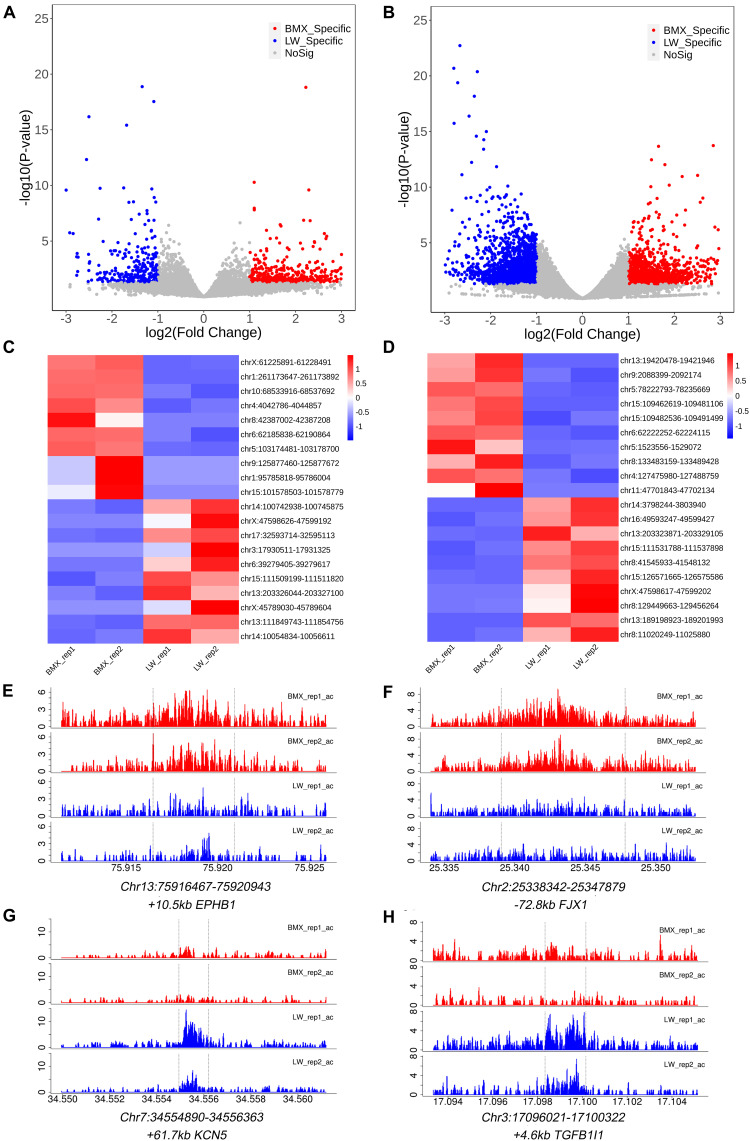
Differential activity analysis on the H3K4me3 and H3K27ac peaks between two breeds and representative validation of differential peaks exhibiting distinct activity of the H3K27ac signal. **(A,B)** Volcano plot showing the differential activity analysis of H3K4me3 and H3K27ac peaks between BMX and LW breeds. **(C,D)** Unsupervised hierarchical clustering of the top differential H3K4me3 and H3K27ac peaks between BMX and LW samples. **(E,F)** The activity track of H3K27ac regions of (Chr13: 75916467–75920943) and (Chr2: 25338342–25347879) with increased hyper-acetylation in the BMX pituitary was related to genes of EPHB1 and FJX1 respectively. **(G,H)** The activity track of H3K27ac regions of (Chr7: 34554890–34556263) and (Chr3: 17096021–17100322) with higher H3K27ac enrichment in LW was related with KCNK5 and TGFB1I1, respectively.

We then sought to identify H3K4me3 and H3K27ac peaks that showed differential activity between the two breeds. At the threshold of fold change ≥ 2 & *P* ≤ 0.05, we identified 793 H3K4me3 peaks (509 and 284 showed higher activity in BMX and LW, respectively) (Figure 3A), and 3,602 H3K27ac peaks showed differential activity between the two breeds (1,614 and 1,918 showed higher activity in BMX and LW, respectively) (Figure 3B). We displayed the activity of the 20 H3K4me3 and 20 H3K27ac peaks that show the most significant differential activity among two breeds (Figures 3C,D).

### Candidate Genes Associated With Breed-Specific Regulatory Elements

We performed gene ontology enrichment analysis on the genes that overlapped with the H3K4me3 peak regions that showed breed-specific activity (334 for BMX and 169 for LW). These genes were enriched in melanosome organization (*P* = 0.004, *AP3B1*, *HPS4*, and *ZEB2*), regulation of T cell differentiation (*P* = 0.018, *ADAM8*, *GTF2H3*, and *SART1*), and activated T cell proliferation (*P* = 0.016, *GPAM*, *RIPK3*, and *SATB1*) (Supplementary Table 2). We displayed two peaks that displayed higher activity in Large White and Bama Xiang pigs. These included a peak (Chr15:111509199–111511820) located at the intron of *PTH2R*, a gene that is expressed in nervous system and plays a role in modulating pituitary functions (Della Penna et al., 2003) (Supplementary Figures 5A,C), and a peak in intron of *ZEB2* (Supplementary Figures 5B,D), which encodes a zinc finger protein that is involved in the regulation of pituitary GH (Kumar et al., 2010). According to published RNA-Seq data, this gene also shows higher expression in the pituitary of Bama Xiang pigs than in Large White pigs (Yang et al., 2018).

For H3K27ac peaks, we found that 951 and 757 genes overlapped with BMX- and LW-specific peak regions, respectively. These genes were enriched in the regulation of growth (*P* = 0.0003, *ABL1*, *ACVR1*, and *BGLAP*), response to GH (*P* = *0.03*, *GHR*, *GHRL*, and *PFDN1*), the thyroid hormone signaling pathway (*P* = 0.0003, ACTG1, MAPK1, and TP53), and the Notch signaling pathway (*P* = 0.01, *ADAM10*, *CDH6*, and *TSPAN5*) (Supplementary Table 3). These results match the function of the pituitary, which secretes GH and thyroid stimulating hormone that regulates the thyroid hormone (Brent, 2012). The Notch signaling pathway regulates endocrine cell specifications in the anterior pituitary (Dutta et al., 2008). We provide examples indicating that the peak (Chr13:75916467–75920943) shows higher H3K27ac activity in BMX, which is located close to *EPHB1* gene (Figure 3E) that plays an important role in the regulation of synapse formation and maturation, migration of neural progenitors, and development of the immune organs (Wei et al., 2017), and locates in the gonadotropes of the pituitary gland, which plays an important role in various niches (Yoshida et al., 2017). Another peak Chr2:25338342–25347879, with higher H3K27ac activity in the BMX, was adjacent to the *FJX1* gene (Figure 3F), which is expressed in telencephalon neurons and has an important function in neuron growth and the differentiation of limbs (Ashery-Padan et al., 1999) and as a direct target of Notch signaling (Rock et al., 2005). Among the H3K27ac peaks that show higher activity in LW than in BMX, we found that Chr7:34554890–34556363 is close to *KCNK5*, which participates in the regulation of platelet size and maturity (Christiansen et al., 2017) (Figure 3G); likewise, Chr3:17096021–17100322 is near *TGFB1I1*, which is involved in male sexual differentiation, cell proliferation, and vascular development (Wang et al., 2011) (Figure 3H) and the regulation of follicle-stimulating hormone in pituitary gonadotropes (Gore et al., 2005). The pituitary regulates the growth and sexual maturation of individuals by secreting GH and gonadotropin (Girard et al., 1999; Themmen and Huhtaniemi, 2000; Kumar, 2016).

### Enrichment of Transcription Factor-Binding Motifs in Breed-Specific H3K27ac Regions

As TF plays an essential role in triggering epigenetic reprogramming (Sindhu et al., 2012), next we investigated the enrichment of TF binding motif in peaks that showed differential activity in H3K27ac between the two breeds using the HOMER program^9^ (Heinz et al., 2010). In total, we found 31 significantly enriched TF-binding motifs (*q* ≤ 0.05) in LW-specific peak regions (Supplementary Table 4), while no significant enrichment was observed in BMX-specific peaks. Although the existence of TF-binding motifs does not guarantee binding of TFs, of these enriched TFs, ATF3 (Hunt et al., 2012) has been linked to brain development and the nervous system; BATF (Williams et al., 2003), JUN (Garcia et al., 2009), and JunB (Szremska et al., 2003) may be related to the immune system, and ESRRG is a sequence motif of a susceptibility gene for obesity-related traits (Dong et al., 2016).

### Diversity of Super Enhancer Among Individuals and Breeds

**FIGURE 4:**
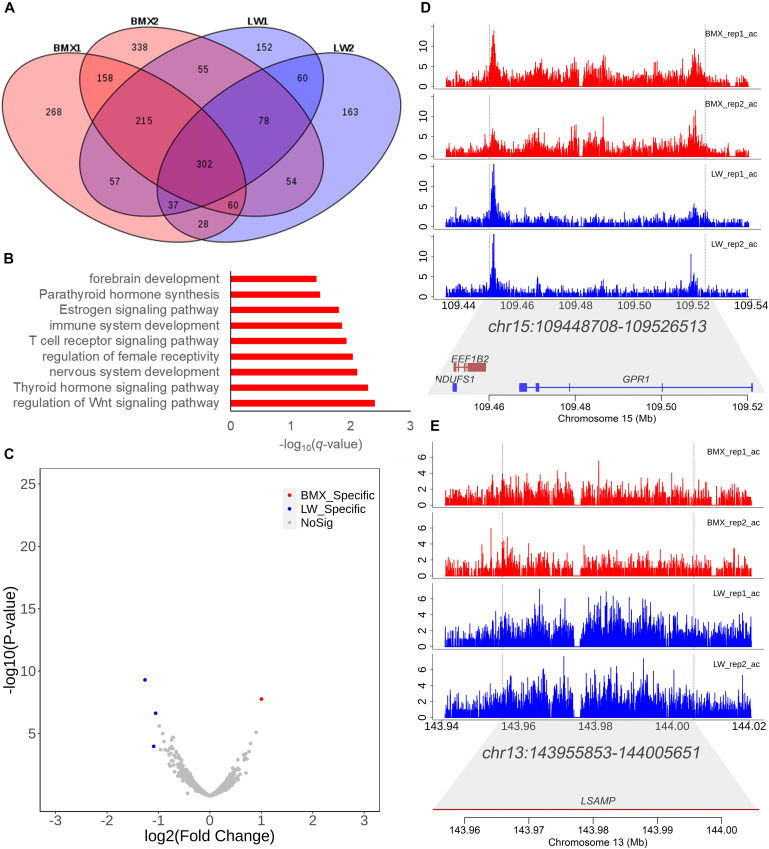
Analysis of super enhancers. **(A)** The distribution of super enhancers in the four individuals. **(B)** Enriched Gene Ontology terms for genes covered by the 302 super enhancers shared cross all samples. **(C)** Volcano plot showing the differential activity analysis of super enhancer between two breeds. **(D,E)** The tracks of H3K27ac activity and gene in the two representative super enhancers that show differential activity between two breeds for reference (Chr15:109448708–109526513 and Chr13:143955853–144005651).

Super-enhancer elements typically include multiple enhancer elements and represent major drivers of transcriptional activation (Lovén et al., 2013). In this study, we identified 1,192, 1,299, 1,017, and 859 super enhancers in four individuals, respectively, using the ROSE algorithm (Whyte et al., 2013). After merging the peaks identified in the four individuals using bedtools, we obtained 2,025 non-redundant peaks. Among these, 302 were shared in all individuals under study (Figure 4A). The genes covered by the 302 super enhancers were enriched in the regulation of the Wnt signaling pathway (*P* = 0.004), central nervous system neuron development (*P* = 0.004), and the thyroid hormone signaling pathway (*P* = 0.01) (Figure 4B and Supplementary Table 5). These pathways match the function of the pituitary. Furthermore, using the DESeq2 program, we identified one and three super enhancers that showed higher activity in Bama Xiang and Large White pigs, respectively (Figure 4C). Chr15:109448708–109526513 and Chr13:143955853–144005651 (Figures 4D,E) are representative examples that display higher activity in Large White and Bama Xiang pigs, and the region of the two super enhancers covers the genes *GPR1*, *EEF1B2*, *NDUFS1*, and *LSAMP*; these can be used as potential targets for neuropsychiatric disorders (Innos et al., 2013).

## Conclusion

To the best of our knowledge, we present the first comprehensive exploration of regulatory elements in the pituitary glands of pigs using ChIP-Seq target H3K4me3 and H3K27ac, two histone modifications with important roles in regulating gene expression. We identified 65,044 non-redundant cis-regulatory sequences, including 23,680 in the H3K4me3 region (putative promoters) and 61,791 in the histone H3K27ac region, which include 12,318 proximal peaks (putative promoters) and 49,473 distal peaks (putative enhancers). It is worth noting that there are 86 H3K4me3 breed-specific regions that overlap with breed-specific regions of H3K27ac. This study enlarges the database of regulatory elements in the pituitary glands of pigs. The identified peaks are valuable for annotating the putative functional mutations identified in genome wide association studies of complex traits in pigs, as well as cross-species comparative studies.

## Data Availability Statement

The ChIP-Seq data and processed data of BMX and LW individuals analyzed for this study can be found at Gene Expression Omnibus (GEO) with accession code: GSE178380 (https://www.ncbi.nlm.nih.gov/geo/query/acc.cgi?acc=GSE178380). The RNA sequencing data of pituitary glands downloaded from three Bama Xiang pigs and three Large White pigs from gsa.big.ac.cn under the GSA number of CRA000876 (https://ngdc.cncb.ac.cn/gsa/browse/CRA000876) (Yang et al., 2018).

## Ethics Statement

The animal study was reviewed and approved by Committee on Animal Biosafety of Jiangxi Agricultural University. Written informed consent was obtained from the owners for the participation of their animals in this study.

## Author Contributions

WL and BY conceived and designed the experiments. ZiZ, ZeZ, and TJ performed the experiments. ZiZ, BY, YZ, TJ, and ZL analyzed the data. YZ, BY, and ZiZ wrote the manuscript. All authors read and approved the final manuscript.

## Conflict of Interest

The authors declare that the research was conducted in the absence of any commercial or financial relationships that could be construed as a potential conflict of interest.

## Publisher’s Note

All claims expressed in this article are solely those of the authors and do not necessarily represent those of their affiliated organizations, or those of the publisher, the editors and the reviewers. Any product that may be evaluated in this article, or claim that may be made by its manufacturer, is not guaranteed or endorsed by the publisher.

## Footnotes

1 https://www.faang.org

2 https://www.ncbi.nlm.nih.gov/geo/query/acc.cgi?acc=GSE178380

3 http://hgdownload.cse.ucsc.edu/goldenPath/susScr11/bigZips/

4 https://ngdc.cncb.ac.cn/gsa/browse/CRA000876

5 https://gat.readthedocs.io/en/Latest/tutorialGenomicAnnotation.html

6 https://david.ncifcrf.gov/home.jsp

7 http://homer.ucsd.edu/homer/motif/motifDatabase.html

8 https://david.ncifcrf.gov/

9 http://homer.ucsd.edu/homer/motif/motifDatabase.html
