# Supplementary material for: Comparative Analysis of Promoters and Enhancers in the Pituitary Glands of the Bama Xiang and Large White Pigs
Source: Front Genet. 2021 Jul 23;12:697994. doi: 10.3389/fgene.2021.697994 (PMC8343535; doi:10.3389/fgene.2021.697994)
Supplement: Supplementary file 2 [file Data_Sheet_2.PDF]

Supplementary Figures

Comparative Analysis of Promoters and Enhancers in Pituitary Gland of Bama Xiang and Large White Pigs

The PDF file includes:

Supplementary figures: Table S1 and Fig. S1 – S5

Table S1 Summary of library complexity parameters for samples investigated in this study.

Sample	NRF	PBC1	PBC2
BMX_rep1_ac	0.972285	0.973153	38.016715
BMX_rep1_input	0.979546	0.980505	51.910762
BMX_rep1_me	0.969492	0.972385	38.551473
BMX_rep2_ac	0.974397	0.974995	40.576606
BMX_rep2_input	0.981653	0.982365	57.227265
BMX_rep2_me	0.977099	0.978777	49.37498
LW_rep1_ac	0.975509	0.976126	42.619897
LW_rep1_input	0.98333	0.983975	63.322858
LW_rep1_me	0.965742	0.96937	35.026429
LW_rep2_ac	0.970491	0.972193	37.501446
LW_rep2_input	0.979451	0.979935	50.251708
LW_rep2_me	0.959149	0.964404	30.967386
Average	0.974012	0.975682	44.612294

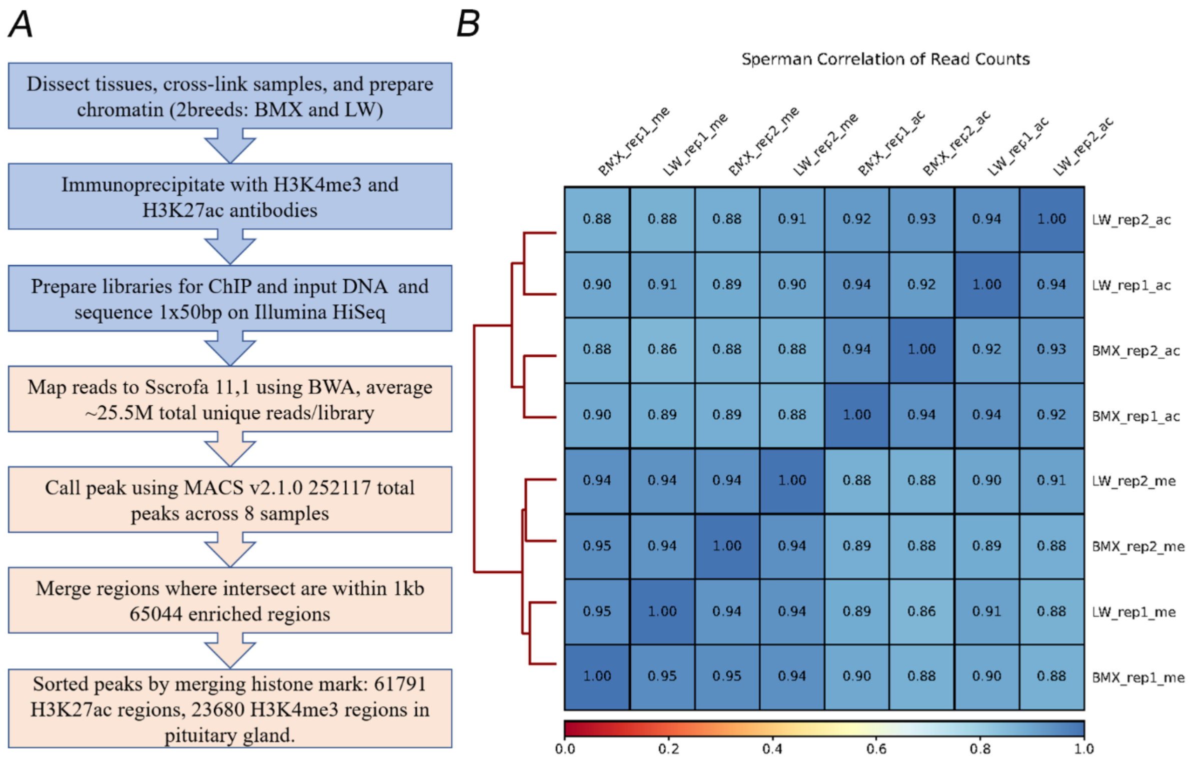

Figure S1. Mapping in vivo promoters and enhancers via ChIP-Seq performed on pituitary of Bama Xiang (BMX) and Large White (LW). (A) ChIP analysis workflow showing experimental (light blue) and computational steps (pink) and summary numbers. (B) The heatmap of lineage-specific histone modification in pituitary across BMX and LW.

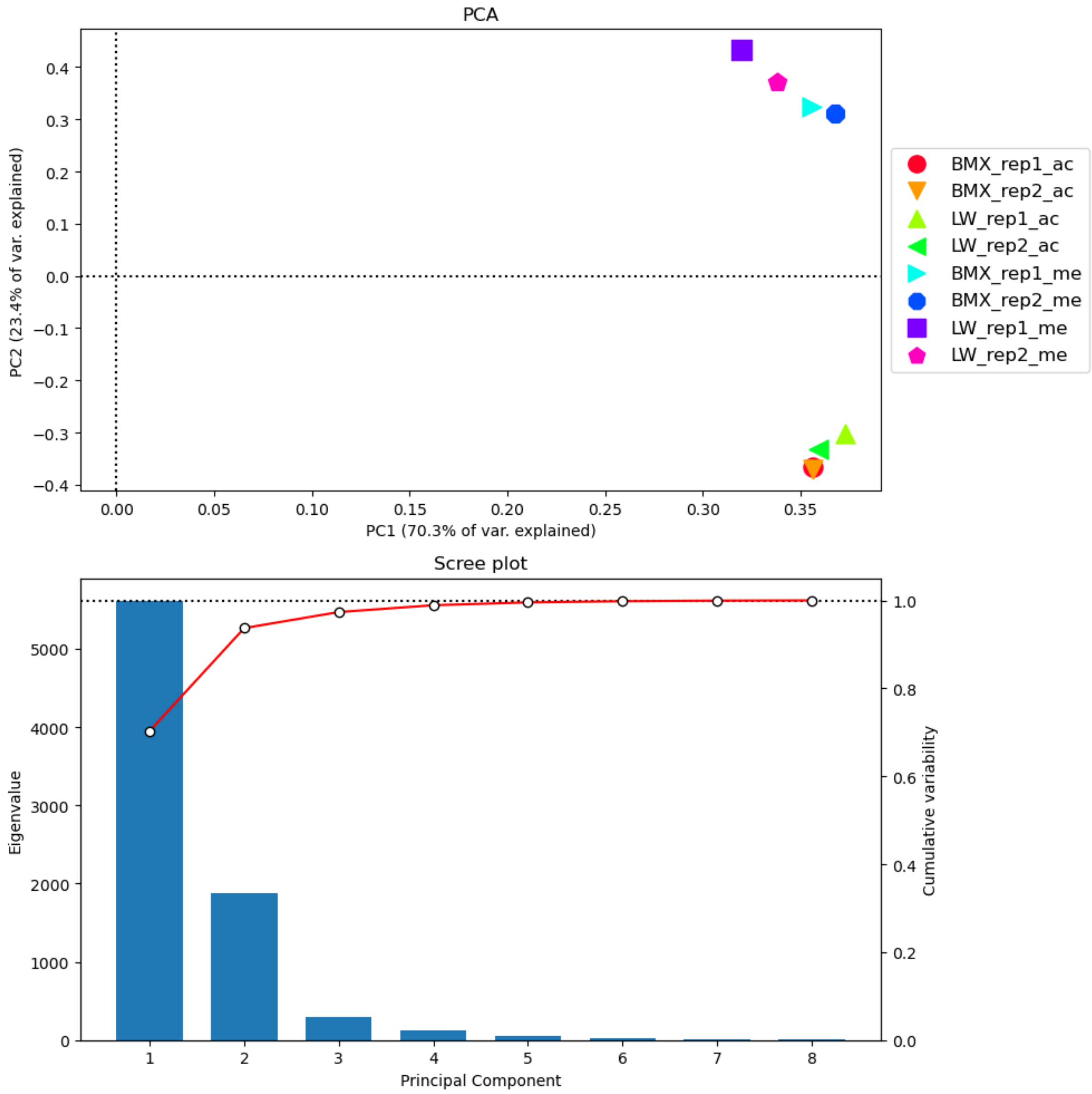

Figure S2. The PCA plot of lineage-specific histone modification in pituitary across BMX and LW.

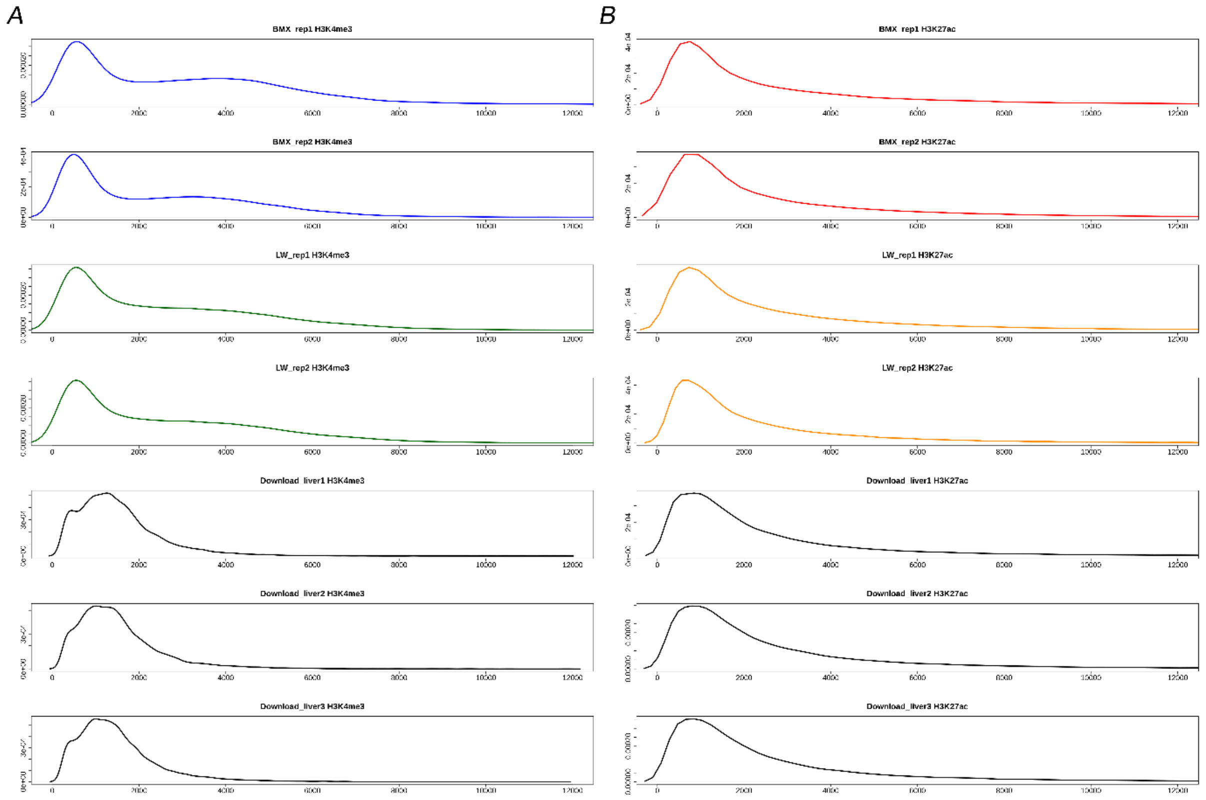

Figure S3. The distribution of length for each ChIP-Seq samples and downloaded liver promoters and enhancers. (A) The distribution of length for each H3K4me3 samples. (B) The distribution of length for each H3K27ac samples. Black represents public data of pig liver promoters and enhancers from Villar et al., 2015.

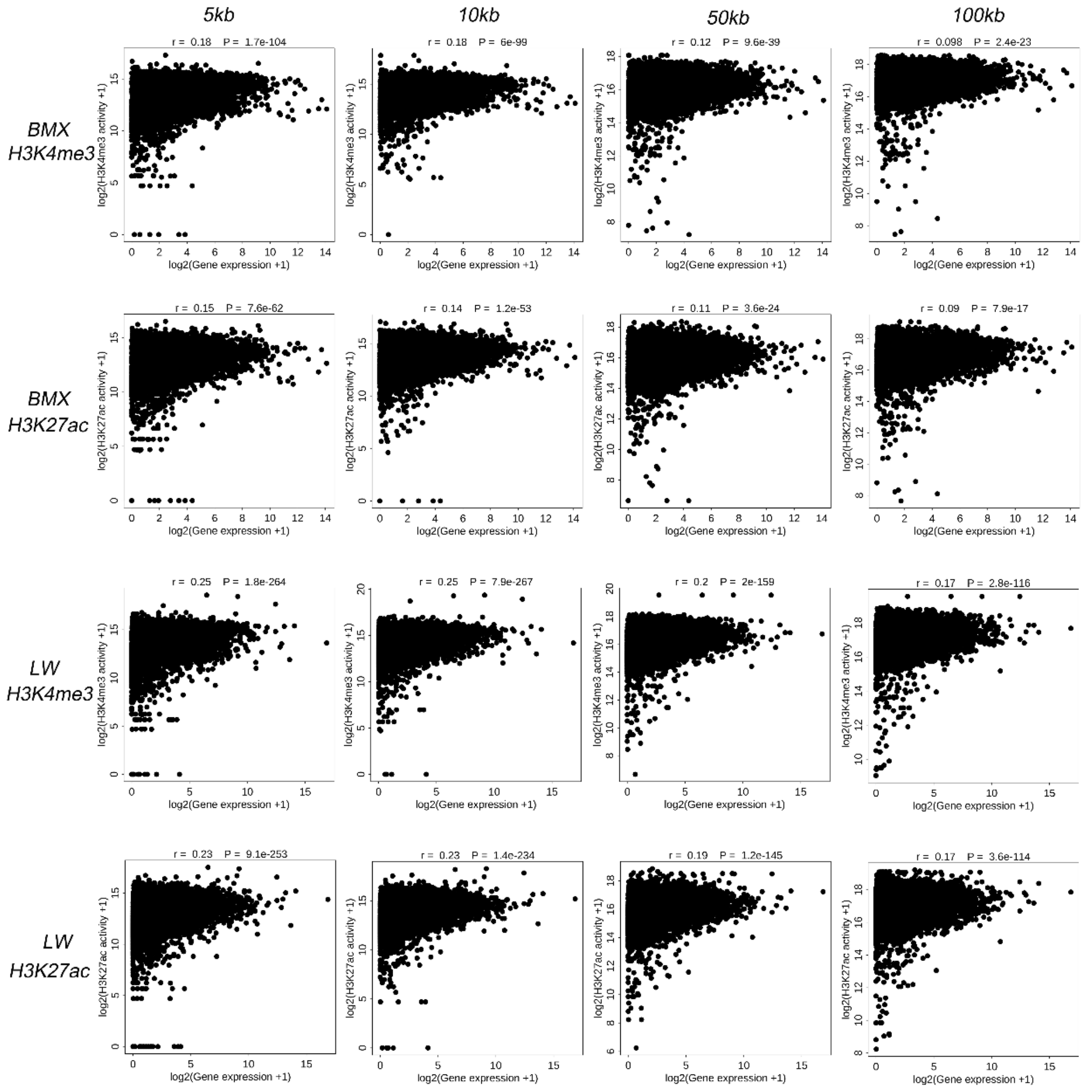

Figure S4. The correlations of expression levels of genes with the H3K4me3 and H3K27ac activity round their TSS regions (e.g., ± 5kb) across the genome despite that ChIP-Seq and RNA-Seq data were generated from different samples

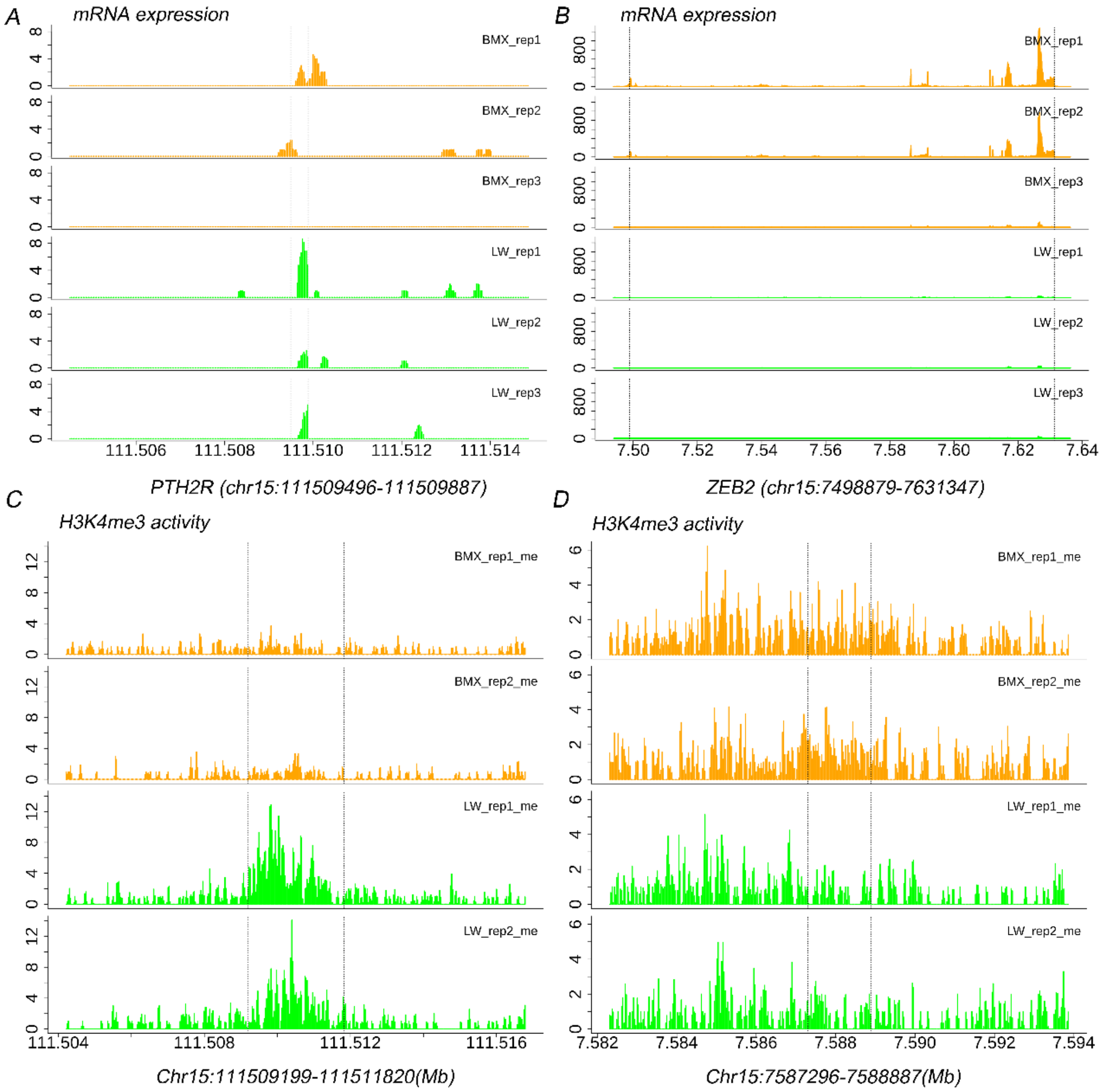

Figure S5. Representative validation of differential peaks exhibiting distinct activity of H3K4me3 signal across BMX and LW pigs. (A) The expression track of LW specific gene PTH2R in pituitary gland of all RNA-seq samples. (B) The expression track of BMX specific gene ZEB2 in pituitary gland of all RNA-seq samples. (C) The activity track of chr15:111509199-111511820 with increased H3K4me3 in the LW pituitary was related to *PTH2R* (chr15:111509496-111509887) in all H3K4me3 ChIP-seq samples. (D) The activity track of chr15:7587296-7588887 with increased H3K4me3 in the BMX pituitary was related to *ZEB2* (chr15:7498879-7631347) in all

H3K4me3 ChIP-seq samples.
